# Job vacancy at the European Centre for Disease Prevention and Control (ECDC)

**DOI:** 10.2807/1560-7917.ES.2025.30.30.20250731

**Published:** 2025-07-31

**Authors:** Eurosurveillance editorial team

**Affiliations:** 1 European Centre for Disease Prevention and Control (ECDC), Stockholm, Sweden

The European Centre for Disease Prevention and Control (ECDC) invites applications for the following position:

**Head of Section AMR and Healthcare-Associated Infections**
The application deadline expires on 16 September 2025.

For more information, please visit the ECDC website: https://www.ecdc.europa.eu/en/about-us/work-with-us/recruitment

